# Interleukin 10 Gene-Modified Bone Marrow-Derived Dendritic Cells Attenuate Liver Fibrosis in Mice by Inducing Regulatory T Cells and Inhibiting the TGF-*β*/Smad Signaling Pathway

**DOI:** 10.1155/2019/4652596

**Published:** 2019-01-17

**Authors:** Yejin Xu, Xinyue Tang, Min Yang, Shengguo Zhang, Shanshan Li, Yukai Chen, Minhui Liu, Yuxiang Guo, Mingqin Lu

**Affiliations:** ^1^Department of Infectious Diseases, Jinhua Municipal Central Hospital, Jinhua, Zhejiang, China; ^2^Department of Gastroenterology, the First Affiliated Hospital of Gannan Medical University, Ganzhou, Jiangxi, China; ^3^Department of Infectious Disease, The First Affiliated Hospital of Wenzhou Medical University, Wenzhou Key Laboratory of Hepatology, Institute of Hepatology, Wenzhou Medical University, Wenzhou, 325000 Zhejiang, China

## Abstract

**Aim:**

To explore the therapeutic effects and mechanisms of interleukin 10 gene-modified bone marrow-derived dendritic cells (DC-IL10) on liver fibrosis.

**Methods:**

In vitro, BMDCs were transfected with lentiviral-interleukin 10-GFP (LV-IL10-GFP) at the MOI of 1 : 40. Then, the phenotype (MHCII, CD80, and CD86) and allo-stimulatory ability of DC-IL10 were identified by flow cytometry, and the levels of IL-10 and IL-12 (p70) secreted into the culture supernatants were quantified by ELISA. In vivo, DC-IL10 was injected into mice with CCl4-induced liver fibrosis through the tail vein. Lymphocytes were isolated to investigate the differentiation of T cells, and serum and liver tissue were collected for biochemical, cytokine, histopathologic, immune-histochemical, and Western blot analyzes.

**Results:**

In vitro, the expressions of MHCII, CD80, and CD86 in DC-IL10 were significantly suppressed, allogeneic CD4^+^T cells incubated with DC-IL10 showed a lower proliferative response, and the levels of IL-10 and IL-12 (p70) secreted into the DC-IL10 culture supernatants were significantly increased and decreased, respectively. In vivo, regulatory T cells (Tregs) were significantly increased, while ALT, AST, and inflammatory cytokines were significantly reduced in the DC-IL10 treatment group, and the degree of hepatic fibrosis was obviously reversed. The TGF-*β*/smad pathway was inhibited following DC-IL10 treatment compared to the liver fibrosis group.

**Conclusion:**

IL-10 genetic modification of BMDCs may maintain DC in the state of tolerance and allow DC to induce T cell hyporesponsiveness or tolerance. DC-IL10 suppressed liver fibrosis by inducing Treg production and inhibiting the TGF-*β*/smad signaling pathway.

## 1. Introduction

Liver fibrosis is the ultimate pathological consequence of chronic hepatic diseases and is featured by the formation and deposition of the extracellular matrix (ECM) [1]. Sustained inflammatory responses lead to liver fibrosis and finally contribute to cirrhosis, portal hypertension, and hepatocellular carcinoma (HCC). Until now, how to effectively prevent hepatic fibrosis and the end-stage liver disease is still an important medical and biological problem to be solved due to the lack of effective drugs and transplant donors for the liver.

Th17 cells participated in the pathophysiology process of inflammatory disease, and the proliferation of Th17 cells leads to the progression of liver fibrosis [2], while regulatory T cells (Tregs) were mainly responsible for the maintenance of immune tolerance and homeostasis by releasing anti-inflammatory cytokines [3]. The imbalance of Th17 cells and Tregs is of vital importance in hepatic diseases, such as autoimmune hepatitis and liver fibrosis [2, 4]. To correct the imbalance of Th17/Treg cells may be a key target for the treatment of liver fibrosis.

Dendritic cells (DCs) are professional antigen-presenting cells (APC) which act as an important role in the activation of primary immune responses, while DCs also silenced T cell immune response and induced Treg development [5]. The functional activities of DCs are mainly depending on their state of activation and maturation; controlling the state of DC maturation is a highly promising therapeutic intervention of diverse diseases. DCs activated by antigen express costimulatory molecules and generate different types of cytokines which determine whether T cells differentiate into Th1, Th2, or Tregs [6]. Genetically modified DCs exhibited tolerance which render them immunosuppressive [7] and may be a promising method to reverse liver fibrosis. Intensive efforts should be taken to design gene therapy strategies for the treatment of liver fibrosis.

IL-10 is acknowledged as an anti-inflammatory and antifibrotic mediator [8]. In addition, IL-10 is also a critical immunoregulatory cytokine which could negatively influence both T cells and DCs. It regulates the function of DCs by reducing the surface markers as well as the secretion of inflammatory cytokines [9]. Previous studies revealed that IL-10-modified DCs display hyporesponsiveness to subsequent lipopolysaccharide (LPS) stimulation, and their allogeneic stimulating capacity is significantly decreased [10]. The TGF-*β*/smad signaling pathway obviously participates in hepatic stellate cell (HSC) activation which plays an important role in the pathogenesis of liver fibrosis [11]. IL-10 is a pleiotropic cytokine characterized by a broad spectrum of anti-inflammatory activities. There seems to be some sort of association between the IL-10 gene and TGF-*β*/smad signaling pathway.

Given the evidence above, we believed that IL-10 is a protective factor in liver fibrosis. Increasing studies researching for the effect of IL-10 gene therapy have been carried out. Considering that IL-10 exerts its influence on the immune system largely by suppressing the function of APCs, we transduced bone marrow-derived DC with lentiviral-interleukin 10-green fluorescent protein (GFP) (LV-IL10-GFP) to explore the changes of DC-IL10 in phenotype and function, effects on the T lymphocyte differentiation, and molecular mechanisms of treatment on liver fibrosis.

## 2. Materials and Methods

### 2.1. Preparation of Bone Marrow-Derived Dendritic Cells

BMDCs were flushed from the femurs and tibiae of 6-8-week-old BALB/c mice. The cells were resuspended at a density of 1 × 10^6^ cells/ml in RPMI 1640 (Gibco, USA) and 10% fetal bovine serum (FBS, Gibco, USA) complete medium with IL-4 (PeproTech, USA, 10 ng/ml) and granulocyte-macrophage colony-stimulating factor (GM-CSF, PeproTech, USA, 20 ng/ml) on the first day. Every other day, the medium was removed, and a fresh medium containing GM-CSF and IL-4 was added.

### 2.2. Lentiviral Transduction of BMDCs

After 6 days of culture period, BMDC populations were transfected with control lentiviral vector GFP (LV-mock-GFP) at multiplicities of infection (MOIs) of 1 : 20, 1 : 40, and 1 : 60 for 72 h to determine the optimal conditions for gene transduction. Transduction efficiency was determined by fluorescence microscopy on day 9 of culture. After the optimum MOI was confirmed, day 6 BMDCs were transfected with LV-IL10-GFP or LV-mock-GFP at a MOI of 1 : 40 for 72 hours to acquire DC-IL10 and DC-mock cells, respectively.

### 2.3. Identification of DC

For immune-phenotyping, 5 × 10^5^ cells (DC, DC-mock, and DC-IL10) were resuspended in 0.2 ml of phosphate-buffered saline (PBS, Thermo Scientific, USA) and incubated with phycoerythrin- (PE-) conjugated murine anti-CD80 and MHCII as well as PE-Cyanine7-conjugated murine anti-CD86, at 4°C for 30 min. All of these antibodies were purchased from eBioscience. Staining with isotype control antibodies was performed in all experiments at the same concentrations as the specific primary antibodies. The fluorescence intensities of the cells were evaluated by flow cytometry, and data was analyzed with FlowJo 7.6.1 software.

### 2.4. ELISA Analysis of the Secretion of IL-10 and IL-12 (p70)

To determine the production of IL-10 and IL-12 (p70) secreted by DC, DC-mock, and DC-IL10 treated with or without 1 *μ*g/ml LPS for 48 h, the supernatants of the DC cultures were collected and the expressions of IL-10 and IL-12 (p70) secreted into the culture supernatants were quantified by ELISA using reagents purchased from eBioscience according to the kit manufacturer's instructions.

### 2.5. Mixed Lymphocyte Assay

T lymphocyte cell proliferation was assessed by mixed lymphocyte reaction (MLR) cultures. Splenocytes were isolated from the cell suspensions by gradient centrifugation at 400 g for 25 min at 4°C with Lymphoprep (TBD Science, LTS1092PK). CD4^+^T cells (4 × 10^6^/ml) were labeled with 5 *μ*mol/l Cell Proliferation eFlour 670 (eBioscience, USA) and then added into a 24-well plate. Allogeneic DC, DC-mock, and DC-IL10 had been pretreated with mitomycin C (Dakewe, Beijing, 25 *μ*g/ml) for 30 minutes and then cocultured with CD4^+^T cells as responders (3 × 10^5^/well) from C57BL/6 mice at ratios of 1 : 5, 1 : 10, and 1 : 30 for 5 days. T cell proliferation reaction was examined by flow cytometric measurement with anti-CD4 mAb (eBioscience, USA).

### 2.6. Administration of DC-IL10 in a Mouse Model of CCL4-Induced Fibrosis

6-8-week-old 18–22 g weighing male BALB/c mice and C57BL/6 mice were purchased from the Shanghai Laboratory Animal Center (Shanghai, China). They were housed under normal laboratory conditions (21 ± 2°C, 12 h light-dark cycle). All experiments were conducted under the standard procedure set by the Committee for the Purpose of Control and Supervision of Experiments on Animals and the National Institutes of Health for the specification use of the experimental animals. The research protocol was approved by the Animal Ethics Committee of Wenzhou Medical University, China. Mice were randomly divided into 4 groups as follows: group 1 (G1, control group), group 2 (G2, model group), group 3 (G3, DC-mock-treated group), and group 4 (G4, DC-IL10-treated group).

Liver fibrosis (G2, G3, and G4) was induced in male BALB/c by twice a week intraperitoneal injection of 0.2 ml/100 g of 40% carbon tetrachloride (CCl4) as a 2 : 3 mixture with olive oil for 8 weeks. The control group was administered with the same volume of olive oil only, twice a week for 8 weeks. In the 6th week of CCl4 injection, the mice in the G3 and G4 groups were treated with DC-mock or DC-IL10 (1 × 10^6^/0.2 ml) suspended in RPMI 1640 into the tail vein (twice a week for 2 weeks) till the 8th week, while the control group and model group received equivalent volumes of RPMI 1640 tail vein injection instead of DC treatment. All mice were sacrificed 72 h following the final CCl4 or olive oil injection at the 8th week, under the condition of anesthesia with 10% chloral hydrate. Blood samples were collected, and liver tissue samples were fixed in 10% buffered formalin or immediately frozen and stored at −80°C for analysis. Splenic tissues were separated for T lymphocyte flow cytometry analysis.

### 2.7. The Differentiation of T Lymphocytes

To analyze the immune-regulatory effect of DC-IL10 on Th17 and Treg cells in CCl4-induced liver fibrosis, the percentages of Th17 and Treg cells in the mouse spleen were measured by flow cytometry. Splenocytes were isolated from the cell suspensions by gradient centrifugation at 450 × g for 25 min at 4°C with Lymphoprep. Following surface staining with anti-CD4-fluorescein isothiocyanate (FITC, 11-0041; eBioscience, San Diego, CA, USA) and anti-CD25-peridinin-chlorophyll-protein (APC, 45-0251; eBioscience), the cells were fixed, permeabilized, and stained with anti-FoxP3-phycoerythrin (PE, 12-5773; eBioscience) to detect the percentage of Treg cells. For Th17 cell detection, the lymphocytes from the spleen were stimulated for 5 h with 50 ng/ml phorbol 12-myristate 13-acetate (PMA), 1 *μ*g/ml ionomycin, 3 *μ*g/ml brefeldin A (BFA), and 1.4 *μ*g/ml monensin (MULTI SCIENCES, China) in RPMI 1640 containing 10% FBS. The cells were harvested and stained with anti-CD4-FITC then fixed and permeabilized with Fix/PERM kit (eBioscience, USA) and stained intracellularly with anti-IL-17-PE (12-7177; eBioscience). Cells stained with IgG isotype control were used as controls. All antibodies were purchased from eBioscience (USA). Data were obtained using a FACSCalibur flow cytometer and analyzed using FlowJo 7.6.1 software [12].

### 2.8. Blood Biochemistry

Blood samples were collected from the retroorbital cavity (~1 ml), and serum was centrifuged by spinning at 3500r for 10 min, after which the samples were stored at −80°C. The levels of aspartate aminotransferase (AST) and alanine aminotransferase (ALT) in the serum were tested by an automatic biochemistry analyzer (Beckman Kurt Co. Ltd., USA).

### 2.9. Measurement of Serum Cytokines

Serum cytokine levels were determined by ELISA according to the manufacturer's instructions. ELISA kits for detecting TNF-*α* and IL-6 were obtained from eBioscience, USA.

### 2.10. Histological Examination

For histopathological study, specimens of the liver tissues were fixed in 4% formalin, embedded in paraffin wax, and sectioned (5 *μ*m). Masson's trichrome staining was used for collagen determination.

### 2.11. Immuno-Histochemical Analysis

For immune-histochemical analysis, 5 *μ*m sections were deparaffnized in xylene and rehydrated in alcohol. A 500 W microwave was used to realize antigen retrieval, and then the sections were heated in citric saline for 15 min. Endogenous peroxidases were blocked by 3% hydrogen peroxide for 10 min. In order to block the nonspecific binding of antibodies, sections were treated with 5% BSA and then incubated with polyclonal rabbit anti-*α*-SMA (ab5694; Abcam, Cambridge, MA, USA) at 4°C overnight in the concentration of 1 : 100. The sections were incubated with HRP-conjugated goat anti-rabbit secondary antibody (1 : 2000; ab7090; Abcam) for 50 min at 4°C after washing in PBS. A DAB enhancer (ZSGB-BIO, ZLI-9018) was used to visualize the positive staining. After that, sections were washed with water before redyeing with hematoxylin.

### 2.12. Western Blot Analysis

Proteins were separated by 10% SDS-PAGE (Solarbio, S1052) and then transferred onto a polyvinylidene difluoride (PVDF) membrane. The membranes were incubated with relevant antibodies in WB primary antibody diluent overnight at 4°C, including TGF-*β*1 (ab179695), *α*-SMA (ab5694), smad2 (ab63576), and smad3 (ab40854), which were purchased from Abcam (Cambridge, MA); anti-Smad7 (sc-11392) which was purchased from Santa Cruz Biotechnology Inc. (Santa Cruz, CA), and rabbit anti-GAPDH (AB-P-R-001) which was obtained from GOOD HERE Technology. Then, membranes were incubated with horseradish peroxide-conjugated secondary antibodies for 1 hour at room temperature after washing. Proteins were visualized by an enhanced chemiluminescence system (Thermo Fisher Scientific Inc., Waltham, MA, USA). Band intensities were analyzed by Quantity One 1-D Analysis software (Bio-Rad Laboratories Inc., Hercules, CA, USA), and values were normalized against the value of GAPDH.

## 3. Statistical Analysis

Statistical analysis was performed by SPSS 22.0. All data (from at least 3 separate experiments) are presented as mean ± standard deviation (SD). Statistical analysis was performed using Student's *t*-test or one-way ANOVA. *p* < 0.05 was considered significant.

## 4. Results

### 4.1. The Confirmation of Optimum MOI

**Figure 1:**
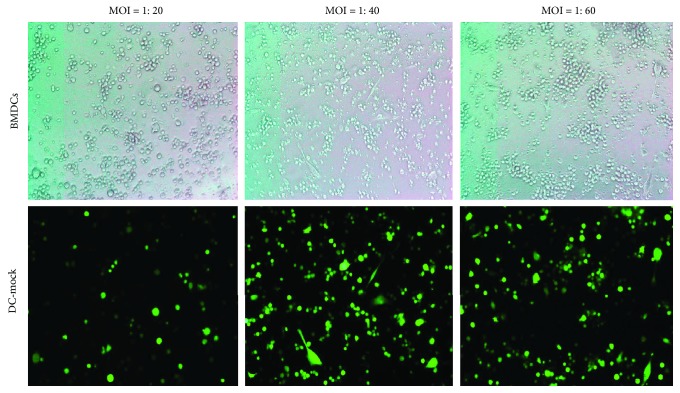
The transfection efficiency analysis of BMDCs. The transfection efficiency can reach up to 80% at the MOI of 1 : 40. Results are from three independent replicates collected on the same day. BMDCs: bone marrow-derived cells; DC-mock: DC transfected with LV-mock-GFP.

BMDCs were transfected with LV-mock-GFP at different MOIs (1 : 20, 1 : 40, or 1 : 60) for 72 h to determine the optimal conditions for gene transduction. The transfection efficiency can reach up to 80% at the MOI of 1 : 40 by fluorescence microscopy, and further elevation of the MOI to 1 : 60 has not increased the fluorescent expression significantly (Figure 1). Hence, we used an MOI of 1 : 40 for LV-IL10-GFP and LV-mock-GFP transfer to BMDCs in the following experiments.

### 4.2. Surface Marker Analysis

**Figure 2:**
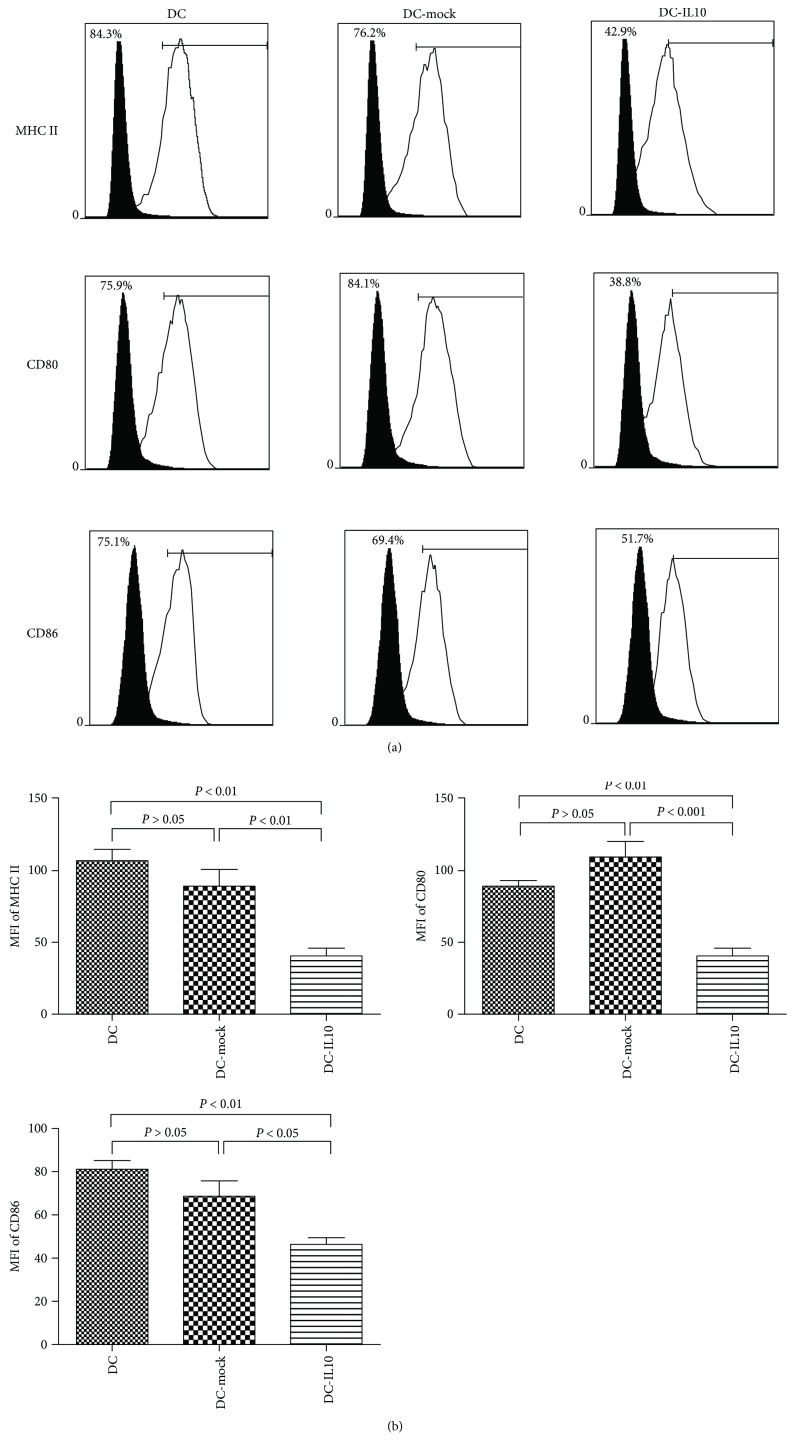
Flow cytometry analysis of surface costimulatory factors of DCs (DC, DC-mock, and DC-IL10). (a) The white histograms indicate frequencies of positively stained cells, and the black histograms represent isotype controls. (b) The MFI of MHC-II, CD80, and CD86 in DC-IL10 was significantly reduced when compared with DC and DC-mock (*p* < 0.05 for all). Results are from three independent replicates collected on the same day and presented as mean ± SD. DC: dendritic cell; DC-mock: DC transfected with LV-mock-GFP; DC-IL10: DC transfected with LV-IL10-GFP; MFI: mean fluorescence intensity.

As depicted in Figure 2(a), the expressions of surface markers MHCII, CD80, and CD86 were significantly increased in both DC and DC-mock populations, while they significantly decreased in the DC-IL10 population. DC-IL10 significantly reduced the mean fluorescence intensity (MFI) of MHC-II, CD80, and CD86 when compared with DC and DC-mock (*p* < 0.05 for all), while there was no statistical significance found in costimulatory molecular expression between DC and DC-mock (*p* > 0.05) (Figure 2(b)).

### 4.3. IL-10 and IL-12 (p70) Quantitation

**Figure 3:**
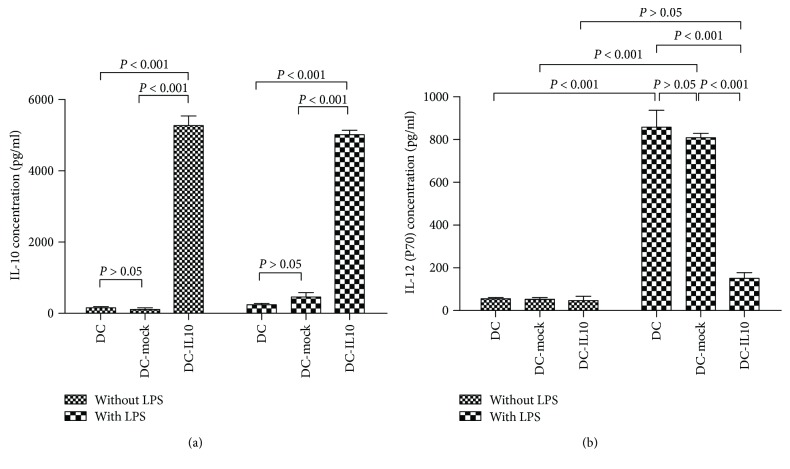
ELISA analyzes the secretion of IL-10 and IL-12 (p70). The DC-IL10 group significantly increased the production of IL-10 (a) and decreased the expression of IL-12 (p70) (b) when compared with DC and DC-mock groups, *p* < 0.001 for all. Results are from three independent replicates collected on the same day and presented as mean ± SD. DC: dendritic cell; SD: standard deviation.

After LPS stimuli for 48 h, the supernatants of the DC cultures were collected, and IL-10 and IL-12 (p70) secreted into the culture supernatants were quantified by ELISA.  Figure 3(a) indicated that IL-10 secreted by DC-IL10 was significantly higher than those by DC and DC-mock either with or without LPS (1 *μ*g/ml) stimuli (*p* < 0.001). The secretion of IL-12 (p70) was significantly increased in DC and DC-mock populations after LPS stimuli (*p* < 0.001), while IL-12 (p70) was significantly inhibited by DC-IL10 when compared with DC and DC-mock populations after LPS stimuli (*p* < 0.001, Figure 3(b)).

### 4.4. Effect of DC-IL10 on T Lymphocyte Proliferation

**Figure 4:**
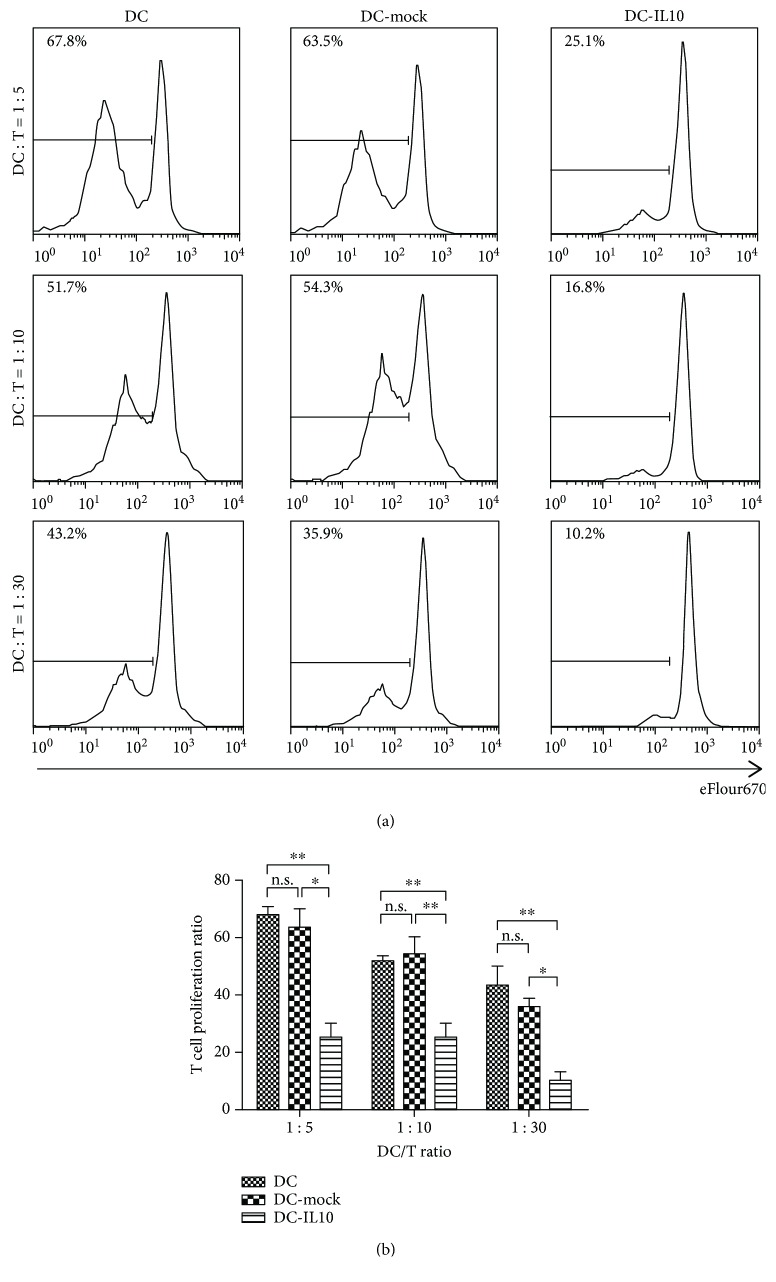
Induction of allogeneic T cell hyporesponsiveness by DC-IL10. (a, b) depicted T cell proliferation histograms after coculture with DC, DC-mock, and DC-IL10. DC-IL10 significantly reduced T cell proliferation when compared to DC and DC-mock (*p* < 0.05 for all). ^∗^*p* < 0.05 and ^∗∗^*p* < 0.01; n.s. indicates *p* > 0.05. Results are from three independent replicates collected on the same day and presented as mean ± SD. *p* values <0.05 were considered to be significant. DC: dendritic cells; SD: standard deviation.

To detect the allo-stimulatory ability of DC-IL10, allogeneic CD4^+^T cell proliferation was detected by FACS with eflour670 stained after 5 days of coculture. DC, DC-mock, and DC-IL-10 were collected and cocultured with CD4^+^T cells at the indicated DC/T cell ratios (1 : 5, 1 : 10, and 1 : 30). As shown in Figures 4(a) and 4(b), DC-IL10 significantly reduced T cell proliferation when compared to DC and DC-mock (*p* < 0.05 for all). DC-IL10 displayed a significantly lower allogeneic T cell stimulatory capacity than did the DC and DC-mock groups. These results suggested that DC-IL10 can induce T cell hyporesponsiveness effectively in vitro.

### 4.5. Effect of DC-IL10 on the Differentiation of T Lymphocytes

**Figure 5:**
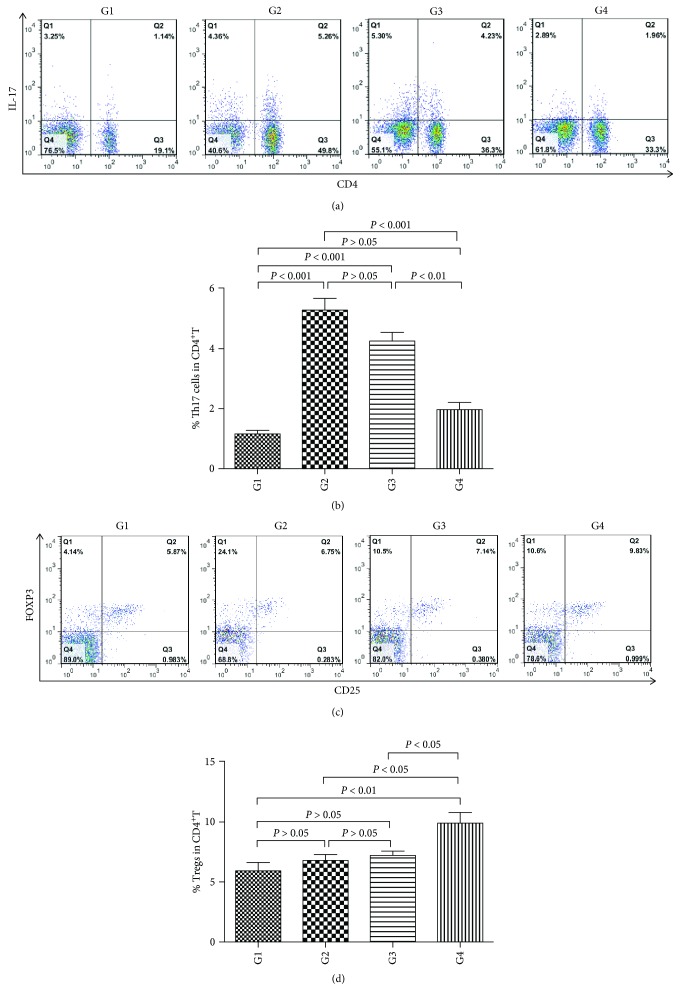
(a) BMDCs were transfected with LV-IL10-GFP or LV-mock-GFP to acquire DC-IL10 and DC-mock cells, respectively. Mice were randomly divided into G1 (control group), G2 (model group), G3 (DC-mock-treated group), and G4 (DC-IL10-treated group). Liver fibrosis (G2, G3, and G4) was induced by twice a week intraperitoneal injection of 40% CCl4 as a 2 **∶** 3 mixture with olive oil for 8 weeks. The control group was administered with the same volume of olive oil only, twice a week for 8 weeks. In the 6th week of CCl4 injection, the mice in the G3 and G4 groups were treated with DC-mock or DC-IL10 (1 × 10^6^/0.2 ml) suspended in RPMI 1640 into the tail vein (twice a week for 2 weeks) till the 8th week, while the control group and model group received equivalent volumes of RPMI 1640 tail vein injection instead of DC treatment. All mice were sacrificed 72 h following the final CCl4 or olive oil injection at the 8th week. Splenocytes were isolated from the spleen in each group of mice to analyze the immune-regulatory effect of DC-IL10 on Th17 cells. The lymphocytes were stimulated for 5 h with PMA and BFA in RPMI 1640 containing 10% FBS and then stained with anti-CD4-FITC. They were fixed and permeabilized with Fix/PERM kit and stained intracellularly with anti-IL-17-PE. The percentages of Th17 cells were determined by flow cytometry. Cells stained with IgG isotype control were used as controls. (b) DC-IL10 suppresses the generation of Th17 cells. The percentages of Th17 cells in CD4^+^T were quantified. Following DC-IL10 administration, the percentages of Th17 cells were significantly decreased when compared with the model group and DC-mock treatment group (*p* < 0.01 for all). Data represent the mean ± SD of 8 mice in each group. G1: control group (*n* = 8); G2: model group (*n* = 8); G3: DC-mock-treated group (*n* = 8); G4 DC-IL10-treated group (*n* = 8). (c) Mice were randomly divided into G1 (control group), G2 (model group), G3 (DC-mock treated group), and G4 (DC-IL10 treated group). Liver fibrosis (G2, G3, and G4) was induced by twice a week intraperitoneal injection of 40% CCl4 as a 2 ∶ 3 mixture with olive oil for 8 weeks. The control group was administered with the same volume of olive oil only, twice a week for 8 weeks. In the 6th week of CCl4 injection, the mice in the G3 and G4 groups were treated with DC-mock or DC-IL10 (1 × 10^6^/0.2 ml) suspended in RPMI 1640 into the tail vein (twice a week for 2 weeks) till the 8th week, while the control group and model group received equivalent volumes of RPMI 1640 tail vein injection instead of DC treatment. All mice were sacrificed for 72 h following the final CCl4 or olive oil injection at the 8th week. Splenocytes were isolated from the spleen in each group of mice to analyze the immune-regulatory effect of DC-IL10 on Treg cells. The lymphocytes from the spleen were surface-stained with anti-CD4-FITC and anti-CD25-APC, then fixed, permeabilized, and stained with anti-FoxP3-PE. The percentages of Treg cells were determined by flow cytometry. Cells stained with IgG isotype control were used as controls. (d) DC-IL10 increases the generation of Treg cells. The percentages of Treg cells in CD4^+^T were quantified. Following DC-IL10 administration, the percentages of Treg cells were significantly increased when compared with the model group and DC-mock treatment group (*p* < 0.05 for all). Data represent the mean ± SD of 8 mice in each group. G1: control group (*n* = 8); G2: model group (*n* = 8); G3: DC-mock- treated group (*n* = 8); G4: DC-IL10-treated group (*n* = 8).

To analyze the immune-regulatory effect of DC-IL10 on Th17 and Treg cells in CCl4-induced liver fibrosis, the percentages of Th17 and Treg cells were measured by flow cytometry. As shown in Figures 5(a) and 5(b), following DC-IL10 administration, the percentage of Th17 cells was significantly lower compared with the model group (G2) (*p* < 0.001), while there was no significant difference between the model group and the DC-mock-treated group (G3) (*p* > 0.05), which indicated that DC-IL10 but not DC-mock can inhibit the ratio of Th17 cells.

Figures 5(c) and 5(d) show that the percentage of Tregs from DC-IL10-treated mice was significantly higher when compared with the model group and DC-mock treatment group (*p* < 0.05 for all). These results revealed that DC-IL10 regulates immune response and alleviates inflammatory response via increasing Tregs and decreasing Th17 cells in CCl4-induced liver fibrosis.

### 4.6. Effect of DC-IL10 on Concentrations of Serum Biochemical

**Figure 6:**
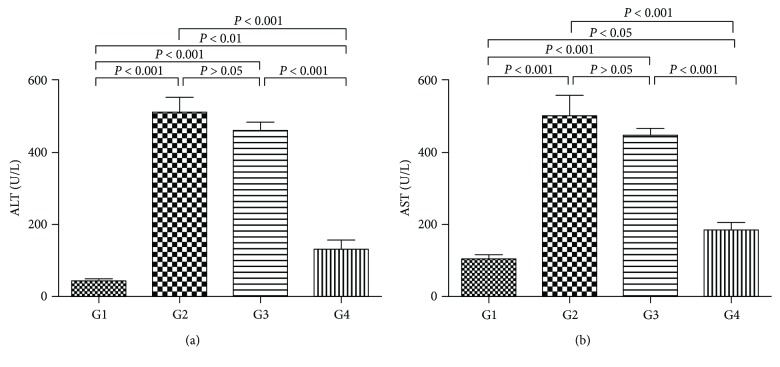
Detection of liver enzyme (ALT and AST) levels. The levels of ALT and AST were significantly decreased in the DC-IL10-treated group compared with the model group and DC-mock-treated group (*p* < 0.001 for all). Data represent mean ± SD of 8 mice in each group. *p* values <0.05 were considered to be significant. G1, control group (*n* = 8); G2, model group (*n* = 8); G3, DC-mock-treated group (*n* = 8); G4, DC-IL10-treated group (*n* = 8). ALT: alanine transaminase; AST: aspartate transaminase.

As shown in Figure 6, the levels of ALT and AST were significantly decreased in the DC-IL10-treated group compared with the model group (*p* < 0.001 for all), while there was no significant difference in the DC-mock-treated group compared with the model group (*p* > 0.05 for all). These results indicated that DC-IL10 infusion can improve liver function.

### 4.7. Effect of DC-IL10 on Liver Inflammation

**Figure 7:**
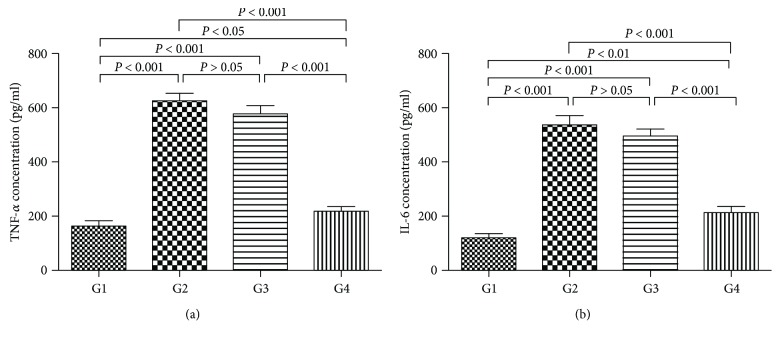
Effect of DC-IL10 on liver inflammation. DC-IL10 treatment significantly reduced the expression of TNF-*α* and IL-6 compared with the model group and DC-mock-treated group (*p* < 0.001 for all). Data represent mean ± SD of 8 mice in each group. *p* < 0.05 was considered to be significant. G1: control group (*n* = 8); G2: model group (*n* = 8); G3: DC-mock-treated group (*n* = 8); G4: DC-IL10-treated group (*n* = 8).

To assess the preventive effects of DC-IL10 on liver inflammation, the accumulation of inflammatory cytokine was measured by ELISA. As shown in Figure 7, higher levels of TNF-*α* and IL-6 were detected in the model group as well as the DC-mock-treated group when compared with the control group (*p* < 0.001 for all), while the DC-IL10 treatment group significantly reduced the above parameters compared with the model group and DC-mock-treated group (*p* < 0.001 for all). According to these results, we hypothesized that DC-IL10 but not DC-mock treatment could attenuate liver fibrosis by reducing proinflammatory factor levels which may relate to the high expression of IL-10.

### 4.8. Effect of DC-IL10 on the Fibrotic Alterations in CCl4-Induced Liver Fibrosis

**Figure 8:**
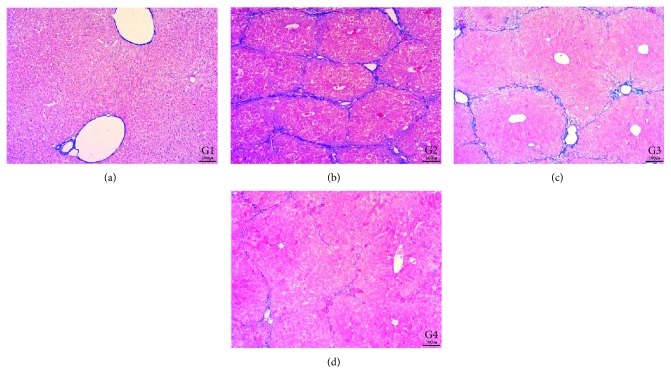
Photomicrograph of a liver stained with MT stain (×100). The control group (G1) and DC-IL10-treated group (G4) showing normal distribution of collagen fibers in the portal areas. The model group (G2) showing marked fibrous bridging with excessive collagen fiber deposition. The DC-mock-treated group (G4) showing slight attenuation of collagen fibers distribution and deposition. G1: control group; G2: model group; G3: DC-mock-treated group; G4: DC-IL10-treated group.

In this study, routine collagen fiber examination of the livers in mice used Masson's trichrome staining. Representative images of the liver morphology are shown in Figure 8. With Masson's trichrome staining, the liver of the control group showed a normal distribution of collagen fibers (G1). The model group (G2) showed severe bridging fibrosis with marked collagen deposition in the liver. In the DC-mock-treated group (G3), collagen deposition slightly decreased, and the collagenous septa became thinner than the model group, while a marked decrease in collagen fibers was present in the group treated with DC-IL10 (G4), which indicated that DC-IL10 can significantly reduce liver damage.

### 4.9. Effect of DC-IL10 on the Activation of HSCs in the Liver

**Figure 9:**
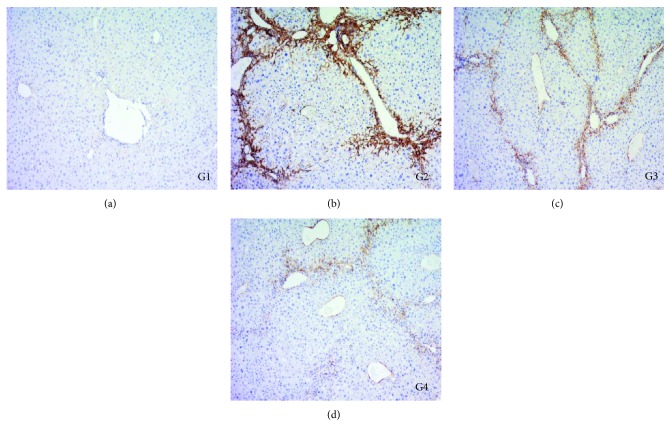
Immune-histochemical analysis of *α*-SMA in liver sections (magnification, ×200). DC-IL10 treatment markedly decreased the *α*-SMA expression compared with the model group and DC-mock treatment group. HSCs: hepatic stellate cells; *α*-SMA: *α*-smooth muscle actin. G1: control group; G2: model group; G3: DC-mock-treated group; G4: DC-IL10-treated group.

*α*-SMA is a sensitive indicator of fibrogenic myofibroblasts [13]. We detected the expression of *α*-SMA in the liver by immune-histochemical staining to evaluate the effect of DC-IL10 treatment on the activation of HSCs in vivo. As shown in Figure 9, only a few cells in the liver section from the control group (G1) were recognized by anti-*α*-SMA. The model group (G2) produced a marked increase in the expression of *α*-SMA as well as the DC-mock-treated group (G3), while the expression of *α*-SMA was significantly reduced following DC-IL10 administration (G4).

### 4.10. Effect of DC-IL10 on the TGF-*β*/Smad Signaling Pathway

**Figure 10:**
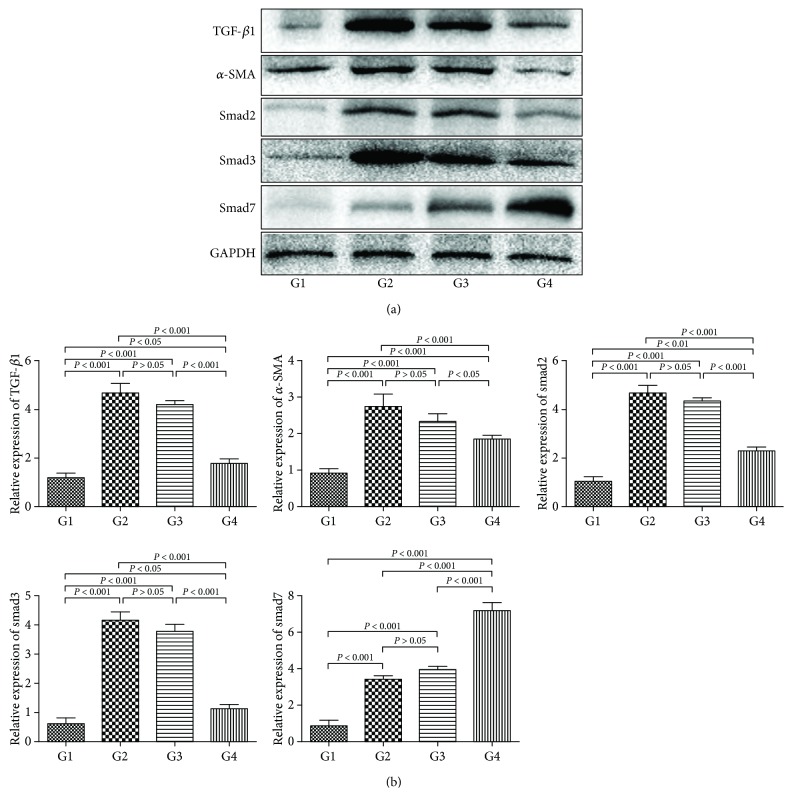
Effect of DC-IL10 on the TGF-*β*/smad signaling pathway. DC-IL10 treatment significantly decreased the expression of TGF-*β*1, *α*-SMA, smad2, and smad3 and increased the smad7 expression than the model group and DC-mock treatment group (*p* < 0.05 for all). Data are normalized to GAPDH protein expression levels. Data represent mean ± SD of 8 mice in each group. GAPDH: glyceraldehyde 3-phosphate dehydrogenase. G1: control group (*n* = 8); G2: model group (*n* = 8); G3: DC-mock-treated group (*n* = 8); G4: DC-IL10-treated group (*n* = 8).

TGF-*β*/smad signaling is a key pathway leading to liver fibrosis [14]. To explore the association between DC-IL10 and the TGF-*β*/smad signaling pathway in liver fibrosis, we examined the protein expression levels of TGF-*β*1, *α*-SMA, smad2, smad3, and smad7 in liver tissues by Western blot (Figure 10(a)). The densitometric quantification (Figure 10(b)) of these bands showed that protein expressions of TGF-*β*1, *α*-SMA, smad2, and smad3 were significantly increased in the model group compared with the control group (*p* < 0.001 for all). However, the DC-IL10 treatment group had markedly lower protein expression levels of TGF-*β*1, *α*-SMA, smad2, and smad3 and higher expression of smad7 than the model group and DC-mock-treated group (*p* < 0.05 for all). Comparing the DC-mock-treated group with the model group, there was no significant difference in the expression levels of the above parameters (*p* > 0.05 for all). These results indicated that DC-IL10 but not DC-mock can suppress the TGF-*β*/smad signaling pathway thereby inhibiting the hepatic fibrosis process significantly.

## 5. Discussion

Hepatic fibrosis is a reversible consequence of chronic liver injury mainly related to the excessive accumulation of ECM proteins in the liver; the risk of cirrhosis could be greatly reduced by early intervention or treatment of liver fibrosis [15]. However, there are no effective drugs to attenuate liver fibrosis presently. New therapy strategies are being intensely investigated. The interaction between hepatic stellate cells/fibroblasts and various immune cells determined the development direction of liver fibrosis. In this study, we made a new attempt to deliver IL-10 gene-modified BMDCs into mice with CCl4-induced liver fibrosis to evaluate the immunotherapy effects and explore the molecular mechanisms of DC-IL10 on liver fibrosis.

As a promising tool for specific cellular therapy, tolerogenic dendritic cells (tDC) play an important role in inducing immunological tolerance in transplantation and autoimmunity [16]. Immature DCs have tolerogenic properties as well, but they are not stable and therefore likely to be unsafe for therapeutic application [17]. DC combined with various immunosuppressive agents can enhance the tolerogenic potential. As immunosuppressive and anti-inflammatory compounds, tDC with low expression of costimulatory molecules are generally characterized by an immature or semi-mature phenotype and produce high amounts of anti-inflammatory cytokines [18].

So far, various projects have been applied to deliver immunosuppressive molecules to DC to regulate immune responses in a precise microenvironment in vivo [19]. IL-10 is a pleiotropic cytokine which related to anti-inflammatory and immunosuppressive properties [20]. In addition, IL-10 is a potential cytokine in the malfunction of DC for the limitation of maturation and reduced capacity to activate Th1 responses, and many researchers have attempted to take advantage of the promising cytokine to improve the therapeutic effect [21]. In our study, we have extended our assessment of the impact of lentiviral delivery of IL-10 on DC phenotype and function both in vitro and in vivo. These new findings have important implications for the potential therapeutic application of IL-10-transduced DC in relation to cell therapy. DC-IL10 exhibited a lower expression of costimulatory molecules (MHCII, CD80, and CD86) than DC and DC-mock, which indicates that IL-10 induces DC with tolerogenic properties in vitro. After LPS stimuli for 48 h, the amount of IL-10 and IL-12 (p70) secreted into the culture supernatants was significantly increased and decreased in the DC-IL10 group, respectively, when compared with the DC and DC-mock groups, which indicates that DC-IL10 exhibits an anti-inflammatory effect by secreting a large amount of IL-10.

TDC regulate peripheral T cell tolerance through inducing immune deviation, incompetence, or apoptosis of effector T lymphocytes, and/or increasing the proportion of Treg [22]. In our study, DC-IL10 significantly reduced T cell proliferation and displayed a significantly lower allogeneic T cell stimulatory capacity in primary MLR than the DC and DC-mock groups. The frequencies of Tregs from the DC-IL10-treated mice were significantly higher when compared with the model group and DC-mock-treated group. The data demonstrate that DC-IL10 induce suppressive Treg and display strong tolerogenic potential, making them suitable for tolerance-inducing therapies.

Regulatory T lymphocytes have been confirmed to play a key role in regulating peripheral tolerance [23]. Previous studies have indicated that the increase in Th17 cells and/or decrease in Tregs is associated with the pathogenesis of hepatic fibrosis [2, 24]. This evidence suggested that the immunosuppressive effect of DC-IL10 on liver fibrosis was related to the balance of Th17/Treg. DC-IL10 significantly reduces the number of CD4^+^ T lymphocytes, but specifically expands the Treg population, thereby regulating cell function. The data from the present study showed that DC-IL10 effectively protected the liver in CCl4-induced hepatic fibrosis, which resulted from a significant increase in Tregs and decrease in Th17 cells.

In addition, the serum levels of ALT and AST as well as the inflammatory cytokine (TNF-*α* and IL-6) in the DC-IL10 treatment group were significantly lower than the model group and DC-mock-treated group. Histological examination and immune-histochemical staining also indicated that DC-IL10 treatment remarkably reduced the liver damage by lessening the deposition of collagen fibers and decreasing the expression of *α*-SMA compared with the model group or DC-mock-treated group. Taken together, these data indicate that administration of DC-IL10 but not DC-mock resulted in a further improvement of liver fibrosis.

One possible reason for the therapeutic effects of DC-IL10 on liver fibrosis might be associated with their capacity to secrete high levels of IL-10 and directly reduce liver inflammation. Previous studies reported that DC-IL10 production of IL-10 is a crucial negative regulator of liver fibrosis [8]. Another reason for the inhibitory effects of DC-IL10 might relate to their ability to induce the expression of Tregs which is involved in the regulation of immune responses. Therefore, preventing the effect of DC-IL10 against liver fibrosis may be mediated by a combined mechanism of anti-inflammatory and immune tolerance. All in all, DC-IL10 may be an effective approach to inducing systemic immune tolerance or hyporesponsiveness against liver fibrosis.

Additional studies have suggested that the imbalance of Treg/Th17 might potentially accelerate liver fibrosis by activating HSCs [25]. These findings indicated that DC-IL10 could inhibit the inflammatory response and the activation of HSCs by regulating the imbalance of Treg/Th17 in liver fibrosis. Inhibiting the activation of HSCs has become a critical treatment strategy for liver fibrosis [26]. Previous investigations have confirmed that the TGF-*β*/smad signaling pathway plays an important role in the activation of HSCs and is considered as a crucial mechanism during the progress of liver fibrosis [27]. In our study, DC-IL10 treatment significantly inhibited the TGF-*β*/smad signaling pathway by reducing the expression levels of *α*-SMA, TGF-*β*1, smad2, and smad3 and increasing the expression of smad7 in the liver and thereby reducing the progress of fibrogenesis. These results indicated that DC-IL10 treatment had an inhibitory effect on HSC activation and hepatic fibrosis which may relate to the production of Tregs and the downregulation of the TGF-*β*/smad signaling pathway.

## 6. Conclusion

IL-10 genetic modification of BMDCs may maintain DC in the state of tolerance and allow DC to induce T cell hyporesponsiveness or tolerance. The inhibitory activities of DC-IL10 on liver fibrosis might involve their capacity to produce high levels of IL-10 and directly inhibit liver inflammation. In addition, DC-IL10 suppressed liver fibrosis by inducing T lymphocytes to differentiate into Tregs and inhibited the TGF-*β*/smad signaling pathway. Infusion of DC-IL10 may be a new approach to treating liver fibrosis. Further studies should be carried out to support our findings. Our current study may provide a foundation for designing gene therapeutics for inhibiting the progression of chronic liver diseases in clinical settings.

## Data Availability

The data used to support the findings of this study are included within the article.

## Abbreviations

IL-10: Interleukin 10
BMDCs: Bone marrow-derived dendritic cells
TGF-*β*1: Transforming growth factor-*β*1
*α*-SMA: *α*-Smooth muscle actin
ECM: Extracellular matrix
Tregs: Regulatory T cells
APC: Antigen-presenting cells
LPS: Lipopolysaccharide
HSCs: Hepatic stellate cells
LV-IL10: Lentiviral-interleukin 10
GFP: Green fluorescent protein
GM-CSF: Granulocyte-macrophage colony-stimulating factor
LV-mock: Control lentiviral
MOIs: Multiplicity of infections
PBS: Phosphate-buffered saline
PE: Phycoerythrin
MLR: Mixed lymphocyte reaction
FITC: Fluorescein isothiocyanate
PMA: Phorbol-12-myristate-13-acetate
BFA: Brefeldin A
FBS: Fetal bovine serum
AST: Aspartate aminotransferase
ALT: Alanine aminotransferase
PVDF: Polyvinylidene difluoride
MFI: Mean fluorescence intensity
tDC: Tolerogenic dendritic cells.
